# The effectiveness of manual stretching in the treatment of plantar heel pain: a systematic review

**DOI:** 10.1186/1757-1146-4-19

**Published:** 2011-06-25

**Authors:** David Sweeting, Ben Parish, Lee Hooper, Rachel Chester

**Affiliations:** 1 Faculty of Medicine and Health Sciences, University of East Anglia, Norwich, Norfolk, NR4 7TJ, UK; 2 Physiotherapy Department, NHS Great Yarmouth and Waveney, 1 Common Lane North, Beccles, Suffolk, NR34 9BN, UK; 3 Physiotherapy Department, Norfolk and Norwich University NHS Hospital Foundation Trust, Colney Lane, Norwich, Norfolk, NR4 7UY, UK

## Abstract

**Background:**

Plantar heel pain is a commonly occurring foot complaint. Stretching is frequently utilised as a treatment, yet a systematic review focusing only on its effectiveness has not been published. This review aimed to assess the effectiveness of stretching on pain and function in people with plantar heel pain.

**Methods:**

Medline, EMBASE, CINAHL, AMED, and The Cochrane Library were searched from inception to July 2010. Studies fulfilling the inclusion criteria were independently assessed, and their quality evaluated using the modified PEDro scale.

**Results:**

Six studies including 365 symptomatic participants were included. Two compared stretching with a control, one study compared stretching to an alternative intervention, one study compared stretching to both alternative and control interventions, and two compared different stretching techniques and durations. Quality rating on the modified Pedro scale varied from two to eight out of a maximum of ten points. The methodologies and interventions varied significantly between studies, making meta-analysis inappropriate. Most participants improved over the course of the studies, but when stretching was compared to alternative or control interventions, the changes only reached statistical significance in one study that used a combination of calf muscle stretches and plantar fascia stretches in their stretching programme. Another study comparing different stretching techniques, showed a statistically significant reduction in some aspects of pain in favour of plantar fascia stretching over calf stretches in the short term.

**Conclusions:**

There were too few studies to assess whether stretching is effective compared to control or other interventions, for either pain or function. However, there is some evidence that plantar fascia stretching may be more effective than Achilles tendon stretching alone in the short-term. Appropriately powered randomised controlled trials, utilizing validated outcome measures, blinded assessors and long-term follow up are needed to assess the efficacy of stretching.

## Background

Plantar heel pain is one of the most commonly occurring foot complaints treated by healthcare professionals [1]. Reliable population based incidence data is lacking in many countries [2]. Within the American population, its incidence has been estimated to be 10% at some point within a lifetime [3] and has been suggested to account for over one million medical visits per annum [4]. It can have a detrimental effect on physical activity, social capacity, mood and vigor [5,6]. Published data estimating treatment and financial costs to the individual and workplace are lacking.

Plantar heel pain is thought to be most commonly associated with the plantar fascia - when the term plantar fasciitis is commonly adopted, but differential diagnosis may include: calcaneal fracture, heel pad atrophy and pain of neural origin [7]. The plantar fascia is a band of fibrous tissue that originates from the medial tubercle of the calcaneus and stretches to the proximal phalanx of each toe [8]. The condition of Plantar Fasciitis is thought to arise from overuse or repetitive micro trauma of the tissue [9]. As the aetiology of plantar fasciitis is unclear, diagnosis is usually based on clinical signs including: plantar heel pain when weight-bearing after a period on non-weight-bearing, pain that eases with initial activity, but then increases with further use as the day progresses, and pain on palpation [1,10,11].

Treatments for plantar heel pain are varied and research findings supporting their use are sometimes conflicting. Stretching is frequently utilised as a conservative treatment for plantar heel pain [1,12]. Systematic reviews investigating the efficacy of conservative treatments for plantar fascia have been published [4,11,13]. However none of the reviews have focused specifically upon stretching. In addition, research investigating the effectiveness of stretching has been published since the searches were performed for these reviews. Indeed the Cochrane review [13] evaluating interventions for plantar heel pain has recently been withdrawn (2010) because it is out of date. There is a need for a rigorous systematic review specifically focusing on the effectiveness of manual stretching as a treatment for plantar heel pain. The objective of this review was to evaluate the effectiveness of stretching compared with no treatment or other conservative treatments on pain and function for people with plantar heel pain. A secondary objective was to identify what type of stretching is most effective in reducing pain and increasing function.

## Methods

### Search strategy

The literature search included the following bibliographic electronic databases: Medline, EMBASE, AMED (all via Ovid), The Cochrane Library and CINAHL (via EBSCO) from inception to July 2010. The search terms used and combined for Medline are detailed in Table 1. Additional searches were undertaken via "clinicaltrials.gov" searching for unpublished trials and via the Physiotherapy Forum "interactive csp" (http://www.interactivecsp.org.uk). Neither of these sources provided any further papers to include in the review. Five hundred and twenty seven potential titles and abstracts were identified from these sources.

**Table 1:** Search strategy used in Medline (Ovid) and run to July 2010

**Database: Ovid MEDLINE(R) In-Process & Other Non-Indexed Citations and Ovid MEDLINE(R) <1950 to Present > Search Strategy**:
1. exp Fasciitis, Plantar/ 2. (plantar* adj5 (heel* or fasciit*)).mp. 3. pain*.mp. 4. 2 and 3 5. ((plantar* adj5 fasciit*) or (spur* syndrome* adj5 (heel* or calcaneal*))).mp. 6. (pain* adj3 heel*).mp. 7. 1 or 4 or 5 8. (stretch* or conservative*).mp. 9. exp exercise movement techniques/or exp exercise therapy/or exp musculoskeletal manipulations/ 10. 18 or 9 11. 6 and 10 12. 7 or 11 13. 1randomized controlled trial.pt 14. controlled clinical trial.pt 15. randomized.ab 16. placebo.ab 17. drug therapy.fs 18. 1randomly.ab 19. trial.ab 20. groups.ab 21. randomised.ab 22. 18 or 15 or 19 or 21 or 14 or 20 or 13 or 16 or 17 23. (animals not (human and animals)).sh 24. 22 not 23 25. 12 and 24

This search was used as the basis of the searches developed for the other databases

### Study selection

Included studies fulfilled the following criteria: prospective controlled trial, investigating adults (over 18 years of age) with plantar heel pain, where stretching (either by the patient themselves, or applied by a therapist but not via a splint or brace) was compared to an alternative intervention or no treatment, published in English, and reporting at least one validated outcome measure, (or measurement by numerical rating scale) relating to pain or function. Studies investigating the effectiveness of stretching applied by splints or bracing, were excluded on the basis that a stretch applied by apparatus over a period of hours was considered a significantly different treatment to stretches applied by the patient themselves or a therapist for a matter of seconds. For inclusion within this review participants needed to either have an explicit diagnosis of plantar heel pain/fasciitis, or fulfill at least two of the following criteria: pain localised to the plantar tissues, localised pain on palpation of the plantar tissues, plantar pain on taking first steps after a period of non-weight-bearing that initially eased but then increased with further use. Both unilateral and bilateral diagnosis or clinical presentations were included. The titles and abstracts resulting from the electronic searches were roughly de-duplicated by loading them onto reference management software (Endnote X4), and then assessed independently in duplicate by two reviewers.

### Data extraction and study quality assessment

Two reviewers independently extracted data from each included study using a data extraction form developed for this review. The completed forms were compared for accuracy and interpretation; where there was disagreement or any ambiguity, both reviewers met to reach agreement. Such disagreements were few in number, but no specific record of them was maintained. If disagreement arose and a consensus could not be reached, the plan was that any disagreement would be settled by further discussion with the third or fourth investigator who would adjudicate if necessary. No disagreements arose which could not be resolved by discussion and always involved clarity of information, sometimes involving the whole team of investigators.

Methodological quality was evaluated via the PEDro (Physiotherapy Evidence-Based Database) scale, (http://www.pedro.org.au). The exact criteria assessed are found in Table 2. Elements were only scored as "yes" where quality clearly met the specified criteria. Where criteria were not met or were unclear, a "no" was scored. Again, this was independently undertaken by two of the reviewers. If disagreement arose and a consensus could not be reached, the plan was that any disagreement would be settled by the third investigator or adjudicator. No disagreements arose which could not be resolved by discussion and always involved clarity of information.

**Table 2:** Results for the modified PEDro rating scale of methodological quality (Item one has been removed from the total score)

The PEDro Scale	DiGiovanni et al [15]	Hyland et al [17]	Porter et al [14]	Radford et al [19]	Sharma et al [20]	Wynne et al [18]
1) Eligibility criteria were specified	YES	YES	YES	YES	YES	YES
2) Subjects were randomly allocated to groups (in a crossover study, subjects were randomly allocated an order in which treatments were received)	YES	YES	YES	YES	YES	NO
3) Allocation was concealed	YES	NO	NO	YES	NO	NO
4) The groups were similar at baseline regarding the most important prognostic indicators	NO	YES	YES	YES	YES	NO
5) There was blinding of all subjects	NO	NO	NO	YES	NO	NO
6) There was blinding of all therapists who administered the therapy	NO	NO	NO	NO	NO	NO
7) There was blinding of all assessors who measured at least one key outcome	NO	NO	NO	NO	YES	NO
9) All subjects for whom outcome measures were available received the treatment or control condition as allocated or, where this was not the case, data for at least one key outcome was analysed by "intention to treat"	NO	NO	NO	YES	NO	NO
10) The results of between-group statistical comparisons are reported for at least one key outcome	YES	YES	YES	YES	YES	YES
11) The study provides both point measures and measures of variability for at least one key outcome	YES	YES	YES	YES	YES	NO
TOTAL SCORE OUT OF 10 (question 1, not included in total score)	4	5	4	8	5	2

### Analysis

Study data were tabulated. Results were assessed to see to whether grouping and meta-analysis would be appropriate. The corresponding author of the three studies which did not provide sufficient data in the text (mean difference between pre and post treatment and standard deviation for each group) [14,17,18] were contacted by email requesting further details. One reply was received [14] but standard deviations were not available.

## Results

**Figure 1:**
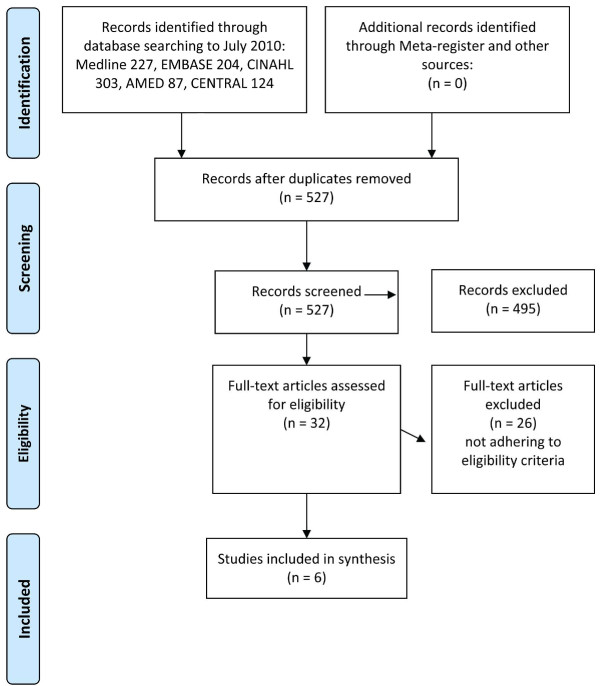
PRISMA flow diagram

Assessment of the 527 titles and abstracts resulting from the searches resulted in exclusion of 495. See PRISMA (Preferred Reporting Items for Systematic Reviews and Meta-Analyses) flow diagram in Figure 1. The remaining 32 were obtained and the full text assessed for inclusion. Twenty-six papers were rejected, as they did not fit the required criteria. A total of six articles were therefore included in this systematic review [14,15,17-20].

### Study characteristics

Five of the six studies utilised a randomised parallel-group design, one of which is described as a pilot study [20] and one study [18] used a "single-blind crossover design". A summary of study and participant characteristics including their clinical signs and symptoms is provided in Table 3. Study quality assessment is summarised in Table 2, and follow up, compliance and details of harmful effects in Table 4. A summary of stretching interventions is provided in Table 5.

**Table 3:** Summary of study characteristics

	DiGiovani et al [15]	Hyland et al [17]	Porter et al [14]	Radford et al [19]	Sharma and Loudon [20]	Wynne et al [18]
Recruitment	Patients with plantar fasciitis not responsive to previous conservative treatment	General Practitioner's surgeries and local gyms	Orthopaedic clinic	Local community (newspaper adverts)	Local community and university	Local community & physician referrals
Clinical signs and symptoms	Maximal pain on palpation of plantar fascia origin. Diagnosis of plantar fasciitis by a Physician	Pain > 3/10 on initial weightbearing. Pain localised at the plantar heel	Pain localised at the plantar heel. Pain at worst on initial weight-bearing	Pain localised at the plantar heel. Pain at worst on initial weight-bearing	Diagnosis of plantar fasciitis by a Physician	Diagnosis of plantar fasciitis
Sample size for each group	A: plantar fascia stretch (non weightbearing) n = 51 B: tendo Achilles stretch (weightbearing) n = 50	A: Stretch (non weightbearing), n = 10 B: Calcaneal taping n = 11 C: No treatment (control) n = 10, D: Sham tape (Control) n = 10	A: tendo Achilles sustained. stretch (weightbearing) n = 54 B: tendo Achilles intermittent stretch (weightbearing) n = 40	A: Calf muscle stretch and sham ultrasound (weightbearing) n = 46 B: Sham ultrasound (Control) n = 46	A: Stretching exercises (plantar fascia and tendo Achilles) n = 8 B: static progressive stretch ankle brace n = 9	A: Counterstrain (non weightbearing) n = 10 B: Placebo non-steroidal anti-inflammatory (Control) n = 10
Mean age (SD, range) in years	A: 44.6 (23-60) B 47.1 (31-60)	A 34.1(5.9), B 45.5 (12.0), C 40.4 (9.4), D 37.6 (10.1)	A 45.4 (11.1) B 45.9 (12.1)	A 50.7 (11.8) B 50.1 (11.0)	A 40.3 (7.0) B 44.2 (11.3)	Mean not documented (20-66)
Symptom duration. Number of subjects and mean duration or range and percentage if unavailable (months)	A: 20 (10-12), 4 (13-18), 1 (19-24), 9 (25-36), 12 (>36). B: 5 (10-12), 15 (13-18), 8 (19-24), 3 (25-36), 5 (>36)	Not documented	A: 54% > 6 B: (53%) > 6	A Median 13 (4-610) B Median 13 (3-121)	A 9.2 (7.7) B 12.2 (6.4)	Not documented
Previous conservative treatment n (%)	Not documented	Not documented	A 19 (35), B 17 (43)	Not documented	"Most"	Not documented
Body Mass Index Mean (SD)	A 28.2, B 28.4	A 26.3 (3.8), B 24.8 (4.4), C 25.4 (4.3), D 23.6 (1.7)	A 27.7 (5.8) B 29.2 (5.6)	A: 31.6 (5.8) B: 32.1 (6.5)	Not documented	Not documented
Hours standing per day Mean (SD)	A: 6, B: 5.4	Not documented	Not documented	A: 7.5 (5.5), B: 9.1 (3.7)	Not documented	Not documented

Abbreviations: SD = standard deviation. Letters A, B, etc refer to group allocation.

**Table 4:** Follow up, compliance and details of harmful effects

	DiGiovanni et al [15]	Hyland et al [17]	Porter et al [14]	Radford et al [19]	Sharma and Loudon [20]	Wynne et al [18]
Follow up (weeks)	8	1 (0 & 1)	4 months (0, 1, 2, 3, and 4)	2 weeks (0 & 2)	12 (0, 4, 8 & 12)	10 (0, 3, intervals to 8-10)
Compliance	Exercise logs provided but not collected for analysis. Questioning: n = 1 in plantar fascia stretch group and n = 4 in Achilles stretch group stopped stretching at 8 weeks	All interventions applied by Therapist	Sustained stretching group: 74.5% (SD 18.4) of stretches completed. Intermittent group: 81.2% (SD 20.6) of stretches completed. *p *= 0.2175	Daily journal kept by all participants. Details of compliance not stated	Not measured.	Not applicable as stretch applied by Therapist.
Drop-outs	Overall 18.8% (n = 19). Plantar fascia stretching group 9.8% (n = 5). Tendo Achilles Stretching group 28% (n = 14).	Overall = 2% (n = 1). Group obtaining the drop-out not specified	Overall 21% (n = 28) Sustained stretching group 6.0% (n = 14). Intermittent stretching group 35.0% (n = 14). Control group 0%	0% (n = 0)	Overall 24% (n = 4), Stretching 12.5% (n = 1), Splint 33% (n = 3)	Overall 5% (n = 1) Crossover trial therefore drop-out not specific to a single group
Reasons for dropping-out	Not stated	Not stated	Requested injection or surgery. Unwilling to travel. Other medical disorders. (no break-down provided)	Not applicable	Not known.	Subject failed to record data fully, results therefore discarded.
Reports of harmful effects	Not stated	No adverse effects from taping. No statement about stretching	Not stated	Stretching group: Increased heel pain (n = 4), Calf pain (n = 4). New lower--limb pain (n = 2). Control group: nil	None reported.	Not stated

**Table 5:** Summary of stretching interventions

	DiGiovanni et al [15]		Hyland et al [17]	Porter et al [14]		Radford et al [19]	Sharma and Loudon (2010)	Wynne et al [18]
Stretching Groups	A	B		A	B			
Type of Stretch	Plantar Fascia. Thumb palpation of Plantar Fascia tension.	Tendo Achilles	Gastrocnemius/ Soleus Plantar Fascia by therapist	Tendo Achilles for 3 minutes	Tendo Achilles for 20 seconds	Tendo Achilles on step	Plantar Fascia stretches and massage. Tendo Achilles stretch	"Counter-strain" in position of 70-80% symptom relief of tender points.
Applied by	Patient	Patient	Therapist	Patient	Patient	Patient	Patient	Therapist
Duration of Stretch	10 seconds	10 seconds	30 seconds	3 minutes	20 seconds	Not Described	30 seconds	90 seconds
Frequency of Stretch	10 reps, 3 × daily	10 reps, 3 × daily	3 reps on day 1, and 3 on day 4	1 rep, 3 × daily	5 reps, 2 × daily	5 minutes daily	3 reps 3× daily	Not described
Weightbearing/Non weightbearing	Non weight bearing	Weight bearing	Non weight bearing	Weight bearing	Weight bearing	Weight bearing	Non weight bearing and weight bearing	Non weight bearing
Knee flexed or extended	Flexed	Extended	Flexed and Extended	Extended	Extended	Not described	Flexed and Extended	Not described
Into/Out of Pain	"To feel stretch"	"To feel stretch"	Not described	Not described	Not described	Not described	Not described	Not described
Supervised?	No	No	Not applicable	No	No	No	No	Not applicable

A total of 365 symptomatic participants, 140 males and 225 females, were included in this review (of whom 269 were allocated to stretching). All studies stated their subject's age, which ranged from twenty-three [15] to sixty-six years [18], mean age in any one intervention group ranged from 34 years [17] to 51 [19]. Four studies recruited participants using methods of convenience such as during scheduled visits to an orthopaedic clinic [14], fliers and advertisements [17,19]. Two studies did not provide details of recruitment [15,20]. The studies varied in duration of follow up from one week [17] to four months [14].

The interventions and comparisons are summarised in Table 5. A variety of stretching techniques were applied in the six studies, with five including tendo Achilles/calf muscle stretches [14,15,17,19,20]. Three papers also included a stretch applied to the plantar fascia, by the patient [15,20] or the therapist [17]. Wynne et al [18] investigated the effectiveness of stretches applied by a therapist to the foot and calf. The precise selection of stretch used by Wynne et al [18], varied from one participant to another based upon the degree of relief it provided to points of local tenderness.

The duration and frequency of stretches varied widely between the studies. Stretching programmes were compared to a range of alternatives including: different stretching techniques, [14,15], calcaneal taping [17], bracing [20], sham ultrasound [19], sham anti-inflammatory tablets [18] and no treatment [17]. The therapist applied stretches directly to the participants in two studies [17,18], while other studies participants were asked to carry out stretches themselves as part of a home exercise programme [14,15,19,20].

Five of the studies measured functional ability using a variety of validated measurement tools; these included the patient specific functional scale [17,21], American Academy of Orthopaedic Surgeon's Lower Limb Core Module, Foot and Ankle Module Questionnaire [14,20,22] the Foot Health Status Questionnaire [19,23] and the Foot Function Index [15,24]. Five studies measure pain as an outcome; two [17,19] using a visual analogue scale, demonstrated to be both valid and reliable [25]. Two studies used the pain subscale of their functional score [15,20], and one [14] did not clearly state how pain was measured; they appear to have extracted questions related to pain from the Foot and Ankle Module Questionnaire. Rather than divide the outcome into pain or function, Wynne et al [18] stated "symptom severity" as a combined score relative to pain, soreness, stiffness and mobility.

### Study quality

The results of the PEDro rating are shown in Table 2. The quality of the studies as determined via the PEDro rating scale ranged from two to eight out of a possible score of ten. Four of the six studies did not document that an intention to treat analysis was used, with three of these studies [14,15,20], not including at least one key outcome measure from at least 85% of participants allocated to each group.

A total of 296 participants were included in the final analyses, with attrition rates from 0% at 2 weeks [19] to 24% at 12 weeks [20]. Larger losses to follow up were noted in studies of longer duration [14,20]. One study reported that there was no loss to follow up [19]. All the other five studies provided numbers for participants lost to follow up, but only two provided reasons [14,18]. Details of numbers lost to follow up are provided in Table 4.

Compliance with treatment regimes was only reported in detail by Porter et al [14] whose sustained stretching group completed 74.5% of their stretches compared to 81.2% in the intermittent group (*p *= 0.218). Radford et al [19] and DiGiovanni et al [15] both asked their participants to keep an exercise log but did not report the results. DiGiovanni et al [15] questioned their participants, and found that one participant in the plantar fascia stretching group and four in the Achilles tendon stretching group had stopped stretching at 8 weeks; reasons were not provided.

### Effectiveness of stretching

Heterogeneity between stretch techniques and comparison groups made meta-analysis inappropriate. The studies were grouped according to the comparison group for stretching: stretching *versus *no treatment, other conservative treatments or alternative methods of stretching. Data has been presented following a narrative review format, noting statistically significant differences. A summary of the results for studies with similar comparators and timescales is provided in Tables 6, 7, 8, 9, 10, and 11.

**Table 6:** Summary of shorter-term changes in mean pain scores comparing groups receiving stretches versus those receiving no intervention or placebo

	Outcome	Group	Baseline score (+/- SD)	Follow up score (+/- SD)	Change in mean score (+/- SD if available)	Between group difference (p value)
Hyland [17]	10 point visual analogue scale	Stretching	6.3 (0.8)	4.6 (0.7)	^#^-1.7	
	at 1 week (0 = no pain)	Control	6.3 (1.2)	6.2 (1.0)	^#^-0.1	Versus stretching 0.026
		Sham taping	6.4 (1.2)	6.0 (0.9)	^#^-0.4	Versus stretching >0.05
Radford [19]	100 mm visual analogue scale	Stretching	70.9 (23.0)	51.1 (29.1)	-19.8 (26.0)	0.138
	1^st ^step pain at 2 weeks (0 = no pain)	Sham ultrasound	75.8 (19.1)	62.5 (29.5)	-13.2 (25.2)	

Abbreviations: SD = standard deviation

^#^Calculated by current authors or estimated from charts

**Table 7:** Summary of shorter-term results for changes in mean functional scores comparing groups receiving stretches versus those receiving no intervention or placebo

	Outcome	Group	Baseline score (+/- SD)	Follow up score (+/- SD)	Change in mean score (+/- SD if available)	Between group difference (p value)
Hyland [17]	Patient Specific Score	Stretching^§^	5.6 (1.1)	4.9 (1.2)	^#^-0.7	0.078
	at 1 week	Control^§^	5.3 (1.5)	4.8 (1.3)	^#^-0.5	
	(10 = full function)	Sham taping	5.3 (0.5)	5.4 (0.6)	^#^-0.1	
Radford [19]	Foot Health Status Questionnaire	Stretching	56.3 (24.5)	72.4 (23.6)	16.2 (19.5)	0.052
	at 2 weeks (100 = full function)	Sham ultrasound	58.2 (24.0)	66.4 (26.2)	8.3 (18.5)	

Abbreviations: SD = standard deviation.

^#^Calculated by current authors or estimated from charts

^§^See main text for discussion regarding the apparent inconsistency in reported *p *values between the stretching and control groups

**Table 8:** Summary of shorter-term results for changes in mean pain scores comparing groups receiving stretches versus those receiving another intervention

	Outcome	Group	Baseline score (+/- SD)	Follow up score (+/- SD)	Change in mean score (+/- SD if available)	Between group difference (p value)
Hyland [17]	10 point visual analogue	Stretching	6.3 (0.8)	4.6 (0.7)	^#^-1.7	0.006
	scale at 1 week	Calcaneal taping	7.0 (0.8)	2.7 (1.8)	^#^-4.3	
Sharma [20]	10 point visual analogue	Stretching	^#^5.3 ^## ^(2.3)	3.5 ^## ^(3.0)	^#^1.75	Not stated
	scale at 4 weeks	Bracing	^#^5.0 ^## ^(0.8)	3.75 ^## ^(2.3)	^#^1.25	Not stated

Abbreviations: SD = standard deviation.

^#^Calculated by current authors or estimated from charts

^## ^Current authors estimation from charts and converting standard error to standard deviation (SD = SE√n)

**Table 9:** Summary of shorter-term results for changes in mean functional scores comparing groups receiving stretches versus those receiving another intervention

	Outcome	Group	Baseline score (+/- SD)	Follow up score (+/- SD)	Change in mean score (+/- SD if available)	Between group difference (p value)
Hyland [17]	Patient Specific Score at 1 week	^§^Stretching	5.6 (1.1)	4.9 (1.2)	^#^-0.7	0.078
	(10 = full function)	Calcaneal taping	4.5 (1.6)	6.2 (1.8)	^#^1.7	
Sharma [20]	AOFAS ankle/hindfoot scale at 4 weeks	Stretching	^#^64 ^##^(15)	^#^65 ^##^(21)	^#^1.0	Not stated
	(100 = full function)	Bracing	^#^64 ^##^(5.7)	^#^65 ^##^(19.8)	^#^1.0	

Abbreviations: SD = standard deviation, AOFAS = American Orthopaedic Foot and Ankle Society.

^#^Calculated by current authors or estimated from charts

^##^Current authors estimation from charts and converting standard error to standard deviation (SD = SE√n)

^§^See main text for discussion regarding the apparent inconsistency in reported *p *values between the stretching and control groups

**Table 10:** Summary of changes in mean pain scores for groups receiving different types of stretches

	Outcome	Group	Baseline Score (+/- SD)	Follow up score (+/- SD)	Change in mean score (+/- SD) if available	Between group difference (p value)
Giovanni [15]	Pain (100 mm visual analogue scale) sub-scale of Foot Function Index (0 = no pain)	Weight bearing Achilles stretch	Not stated	Not stated	Pain at worst -14.7 ^##^(+/-19.9) 1^st ^am steps -13.2 ^##^(+/-27.7) Combined pain score -13.0^##^(+/-20.8)	Pain at worst p = 0.02 ^#^Mean 11.3 1^st ^steps in morning p = 0.006 ^#^Mean 17.9 Combined score p > 0.05 ^#^Mean 6.0
		Non weight bearing plantar fascia stretch	Not stated	Not stated	Pain at worst -26.0 ^##^(+/-24.3) 1^st ^am steps -31.1 ^##^(+/-28.8) Combined pain score -19.0 ^##^(+/-19.9)	
Porter [14]	Foot and ankle pain score	Sustained	57.5 (20.1)	79.7 (17.5)	^#^22.2	P = 0.315
	(100 = no pain)	Intermittent	53.5 (22.0)	82.5 (15.2)	^#^29.0	

Abbreviations: SD = standard deviation, SE = standard error.

^#^Calculated by current authors or estimated from charts

^##^Current authors estimation from charts and converting standard error to standard deviation (SD = SE√n)

**Table 11:** Summary of changes in mean functional scores for groups receiving different types of stretches

	Outcome	Group	Baseline score (+/- SD)	Follow up score (+/- SD)	Change in mean score (+/- SD) if available	Between group difference (p value)
Giovanni [15]	Function Index	Weight bearing, Achilles stretch	Not stated	Not stated	-8.3 ^##^(16.6)	0.058
	(0 = full function)	Non weight bearing, plantar fascia stretch	Not stated	Not stated	-19.6 ^## ^(18.7)	
Porter [14]	Foot and ankle function score	Sustained	68.8 (19.9)	82.5 (18.7)	^#^13.7	
	(100 = full function)	Intermittent	62.3 (19.7)	88.5 (14.2)	^#^26.2	>0.05

Abbreviations: SD = standard deviation, SE = standard error.

^#^Calculated by current authors or estimated from charts

^**##**^**Current authors estimation from charts and converting standard error to standard deviation (SD = SE√n**)

### Harms

Three papers [17,19,20] provide details of the presence or absence of a harmful effect of their interventions; see Table 4. Hyland et al [17] reported no harmful effects from taping, but made no statement with regards to stretching. Sharma and Loudon [20] report no harmful effects. Radford et al [19] reported adverse effects in 10 participants within the stretching group. These effects included increased pain in the heel, calf and other areas of the lower limb. There were no adverse effects reported from the control group.

### Pain and function - stretching versus no intervention

Three studies compare stretching with no treatment [17] or a placebo intervention [17-19]. Incomplete data prevented meta-analysis. Wynne et al [18] did not produce independent results for pain and function, but rather grouped them as "symptom severity".

Both Hyland et al [17] and Radford et al [19] reported improvements in pain over time in the stretching groups; reported as statistically significant (p < 0.001) in the Hyland et al trial (Table 6). However improvements were also demonstrated in control groups, indicating a strong placebo or non-intervention effect. Hyland et al [17] demonstrated that in comparison to no treatment, the stretching group obtained greater pain relief (*p *= 0.026). However, this same stretching group reported no difference in pain relief than a group receiving sham taping (*p *> 0.05). The study with the highest quality rating on the modified PEDro scale, [19] found no significant difference in pain relief between stretching and a control intervention of sham ultrasound (*p *= 0.138).

Neither Radford et al [19] or Hyland et al [17] reported a statistically significant change in the functional ability of the participants after completing the stretching intervention, (Table 7). It should be noted however, that the data published by Hyland et al [17] shows the mean function of the stretching group to have declined to a greater degree than the control group; who are themselves described as having a statistically significant decline in function (*p *= 0.003). Radford et al [19] reported that both the stretching and control groups improved over time with a small improvement in favour of the stretching group, but this was not statistically significant (*p *= 0.052).

Wynne et al [18] report an improvement in symptom relief, (combined score of pain, soreness, stiffness and mobility) in both stretching and control groups immediately following treatment, which consistently reached statistical significance in the stretching group (*p *< 0.05) but only after the first of three treatments in the control group. There was a statistically significant difference between groups, in favour of the stretching group two days post treatment but the authors report that this was not maintained. Results from this study were difficult to interpret. However our observations of charted data was that participants in both groups reported similar or worse symptom severity prior to their third treatment than prior to their second.

### Pain and function - stretching vs another conservative treatment

Two studies compared stretching with another treatment. Hyland et al [17] found that stretches were less effective than calcaneal taping in reducing pain (*p *= 0.006). Sharma and Loudon [20] demonstrated that stretching or bracing may both reduce pain over time (*p *< 0.05), however no group differences were demonstrated in reducing pain on the Foot Function Index (*p *= 0.77) or morning pain (*p *= 0.79). Within their study any reduction in pain due to stretching appears to occur in the first month [20] (Table 8).

There was no statistically significant difference between groups in either study in terms of improvement in function (Table 9). Our observation of Hyland's data [17] indicates an improvement in function in the taping group, and a slight decrease in function in the stretching group; reported by the authors as statistically insignificant. Both groups in Sharma and Loudon's study [20] improved over time (*p *= 0.005). Observation of their data indicates a greater improvement in function in the bracing group one month after completing treatment [20]. However, Sharma and Loudon [20] used the American Orthopaedic Foot and Ankle Society Ankle-Hindfoot scale, which incorporates function as just one component of this outcome measure, and this may not therefore be a true representation of function alone.

### Pain and function - comparing two types of stretching

One study [15]) compared different stretching techniques. See Tables 10 and 11. DiGiovanni et al [15] compared non-weight-bearing plantar fascia stretches with weight-bearing tendo Achilles stretches. Both groups reported a statistically significant reduction in pain from baseline to 8 weeks (Table 10). On comparing the two groups, a significant reduction in two of seven aspects of pain was reported; pain "at its worst" (*p *= 0.02) and on "first steps in the morning" (p = 0.01) was reported in the group carrying out plantar fascia stretches versus Achilles tendon stretches at the eight week follow up. There was a similar trend towards improved function in the plantar fascia stretching group compared with Achilles tendon stretches at eight weeks (see Table 11), but this did not reach statistical significance (*p *= 0.058).

Porter et al [14] compared 3 minute sustained stretches with 20-second intermittent tendo Achilles stretches (Tables 10 and 11). Both groups improved in terms of pain and function at each of four monthly follow up periods. There were, however, no statistically significant differences between groups for pain (*p *= 0.315). With regards to function, Porter did report a statistically significant difference in favour of the intermittent stretching group when analysed using mixed-model repeated measures ANOVA (*p *= 0.015). This was visually evident to the reviewers in terms of both pain and function; we observed a trend in favour of intermittent stretches, with the most rapid improvement occurring in the first month. However, pair-wise comparison of the two groups did not show any statistically significant difference at any one time point.

## Discussion

The results of this systematic review demonstrate that patients with plantar heel pain who stretch tend to improve over time with regards to both pain and function, but when stretching is compared to other interventions, including sham treatment, no statistically significant benefit was observed. In comparison to no intervention, one study [17] demonstrated that stretching was statistically significantly more effective in reducing pain, although the clinical significance is debatable. The study gaining the highest PEDro quality rating [19] did not find stretching to be any more beneficial than a control intervention. However, the type of stretching may be relevant - DiGiovanni et al [15] compared different stretching techniques, and found stretching of the plantar fascia in non weight bearing, to be significantly more effective than tendo Achilles stretching in weight bearing in reducing some, but not all aspects of pain at eight week follow up.

Previous reviewers [11] and authors of clinical guidelines [1], included just two of the studies in this review [14,15], and concluded that there is some, scientific evidence described as moderate quality [1], and poor quality [11] to support the use of stretching for the treatment of plantar heel pain in terms of short term relief. Landorf and Menz [4] included two primary studies in their review [15,26] only one of which [15] fulfilled the inclusion criteria for this current review. They concluded that the available evidence was inadequate to support stretching exercises as being any more effective than other interventions or no intervention in the treatment of plantar heel pain. Following our review of six papers, we would support Landorf and Menz's findings [4] that at present there is insufficient evidence to draw any conclusions about the comparative effectiveness of stretching.

The relatively small number of participants evaluated in most of the studies may have influenced the results of this review. Although there was a trend for an improvement in participants who underwent stretching, only one study [17] demonstrated a statistically significant difference between stretching and a control treatment. The study with the highest PEDro quality rating [19] did not find their stretching programme to be any more effective than sham ultrasound. This was the only study to report the use of a power calculation in selecting their study sample size. Other studies, in particular those with smaller samples, may have suffered from a type II error in which potential differences between groups are not detected due to inadequate power. It therefore remains unclear whether stretching exercises are more effective than other treatments or no treatment in the management of plantar heel pain. We recommend that sample sizes for future studies are pre-specified and based on appropriate power calculations.

It is important to note the difference between statistical significance and clinical significance [27]. The only study demonstrating a statistically significant difference between stretching and a control treatment [17], used a visual analogue scale evaluating pain on first steps in the morning, and reported a mean improvement in the stretching group of 1.7 on a scale of 0-10. Research has recently been undertaken evaluating a similar scale [28], and it was concluded that the minimal important difference in score required for a patient with plantar heel pain to perceive benefit from treatment, was an improvement of 19 mm on a 100 mm scale. On this basis, the clinical significance of the improvements demonstrated by Hyland et al [17] can be questioned.

The length of follow up time varied from 1 week [17] to 4 months [14]. This has the potential to influence the results and other factors such as dropout rates. This influence may be reflected in the results; the study with the shortest follow up time was the only one to report a statistically significant benefit to stretching in comparison to a control or other intervention and had a drop out rate of only 2%. In comparison, the studies with the longest follow up periods [14,20], reported results that were not statistically significant, and had the highest dropout rates of 21% and 24% respectively (see Table 4).

Subject characteristics may have played a role in response to treatment. The duration of symptoms varied between and within studies. In one paper, this ranged from 3 to 121 months [19]. Other chronic conditions such as back pain have been shown to be less likely to respond to treatment [29], and this variation may have an impact on the success of any intervention. Research investigating the influence of the duration of plantar heel pain on its responsiveness to treatment, may therefore be helpful to those evaluating the effectiveness of treatment modalities in the future.

The specific anatomical structure under stretch may have influenced the effectiveness of the technique. One study [15] compared two different stretches (plantar fascia stretches and tendo Achilles stretches). A significant reduction in pain "at its worst" (*p *= 0.003) and on "first steps in the morning" (*p *= 0.01) was reported in the group carrying out plantar fascia stretches in comparison to tendo Achilles stretches at eight weeks. The only paper to show a statistically significant benefit from stretching over a control intervention [17] used a plantar fascia stretch in combination with a stretch to the calf muscles. The highest quality study [19] did not find any benefit from a tendo Achilles stretch in isolation when compared to a control intervention. This may suggest that in the short term at least, plantar fascia stretching is more effective than tendo Achilles stretching in isolation.

There was considerable variation in the frequency of the stretching techniques applied (Table 5). This factor alone may have influenced results and makes direct comparison difficult. The one study that found a statistically significant benefit from stretching in comparison to a control group [17] did the least number of stretches (two sessions in a week). Other studies [14,15,19,20], however, did also demonstrate some improvement in pain compared to alternative or control interventions, but these improvements were not statistically significant. Therefore, the available evidence does not allow any firm conclusions to be made regarding the optimal frequency of stretches.

There was a wide variation in the duration of stretch applied, ranging from ten seconds [15] to three minutes [14]. One study [14] specifically compared sustained stretching for three minutes with intermittent stretching for twenty seconds and found no statistically significant difference between the two groups, although the potential for significant difference in terms of increased function was reported using the mixed method repeated ANOVA. The position that the stretches were performed in also varied. In two studies the participants stretched in a weight-bearing position, in two they were in a non-weight bearing position and in another study one intervention group was weight bearing and one was non-weight bearing [15]. Although the non-weight bearing stretch did show some significant improvements over the weight bearing stretch in the latter study, the difference in the anatomical structure being stretched (plantar fascia versus tendo Achilles) limits any conclusions in this respect. It appears that no clear conclusions can be drawn regarding the most effective stretch duration, or position.

Another source of variation was the number of repetitions that the participants in each study were asked to perform. The highest number of repetitions was 210 per week [15], and the lowest number was 6 per week [17]. This is clearly a wide range, and is likely to have an influence on the outcome. However, whilst the study with the lowest number of repetitions found a statistically significant difference, the study with the highest repetitions [15] also found some improvements in pain scores. Obviously no clear conclusions can be drawn regarding the optimum number of repetitions.

In four of the studies [14,15,19,20], the participants implemented the stretches themselves; in the two additional studies the therapist applied the stretch [17,18]. The highest quality study [19] used self-applied stretching and found it no more effective than a control intervention. No studies compared self-stretches with therapist-applied stretches and this is an aspect that may benefit from further research and cost benefit analysis.

Four studies used a visual analogue scale for measuring pain, [15,17,19,20] and demonstrated that between 1 week and two months of commencing a stretching programme there is a decrease in pain, although the effectiveness of stretches in reducing pain may not be above that of a control group [17,19] or alternative treatment [17,20]. DiGiovanni et al [16] did carry out a two year case series in which participants from the Achilles stretching group, joined participants from the plantar fascia stretching group in carrying out plantar fascia stretches for a further two years. Although an improvement in pain relief continued, the absence of a control group limited any conclusions that could be drawn about the benefit that might be gained from continuing to stretch for a longer period. Also as plantar heel pain may be self-limiting [4], the continued improvement described by DiGiovanni et al [16] may simply represent the natural history of the disorder.

Four studies measured functional ability [14,17,19,20] as an outcome after stretching. Porter et al [14] reported a potentially significant improvement in functional ability, but not pain at monthly follow up periods for 4 months in favour of intermittent 20 second stretches versus sustained 3-minute stretches. Their study did not include a control or alternative treatment group. The study with the highest methodological quality rating using the PEDro score [19], found a trend (*p *= 0.052) in favour of the stretching group over the control group for the function sub-scale of the Foot Health Questionnaire at two week follow up. Sharma and Loudon [20] used the American Orthopaedic Foot and Ankle Society Ankle-Hindfoot scale, and found significant improvements 4 weeks after completion of an 8 week stretching programme (*p *= 0.005), but function is just one of component of this outcome measure and there was no difference between this and the results from a group using bracing (*p *= 0.78). Conversely, Hyland et al [17] showed a negative trend with regards to function at one week follow up in the stretching group. This negative trend might be as a result of the shorter follow up time utilised by Hyland et al [17]; which might not be an adequate period to detect functional changes. However, as previously mentioned, although described as not statistically significant, the data published by Hyland et al [17] shows the mean function of the stretching group declined to a greater degree than the control group; who were themselves found to have experienced a significant decline in function (*p *= 0.003). Whilst pain relief is likely to be a primary goal of treatment for people suffering from plantar heel pain, improvement in function is equally crucial. With this in mind, an important question was unable to be fully answered by this review.

Only six eligible studies were retrieved. Having such a small number of studies within a systematic review may lead to misleading conclusions. This does highlight the need for further adequately powered randomised controlled trials. The internal validity of future studies would be enhanced by allocation concealment and blinding of assessors. Future studies should also include presentation of mean differences and measures of variability, (e.g. standard deviations or 95% confidence intervals) in outcome scores pre and post treatment for each group. This would allow results to be attributed to the intervention rather than the passage of time and allow meta-analysis.

Certain limitations of this review must be acknowledged. Firstly the search strategy relied exclusively on computer databases and no hand searches were undertaken, thus relevant papers may have been missed. Although the search attempted to identify unpublished research, it is possible that some relevant pieces of grey literature (such as university theses) were not uncovered. Any exclusion of unpublished work that may subsequently have occurred increases the possibility of reporting or publication bias. This review only included papers published in English. This again may have resulted in the exclusion of relevant research. Appropriate caution should therefore be applied when interpreting the results of this systematic review.

## Conclusions

It cannot be stated from the currently available evidence that stretching is any more effective than other interventions or control groups in relieving plantar heel pain. The main pain-relieving benefits of stretching appear to occur within the first two weeks to four months. There is no conclusive evidence regarding the most effective number of repetitions or frequency of stretching, or whether self or therapist applied stretches are most effective. Inclusion of stretches directly to the plantar fascia may provide better short-term pain relief than stretching the tendo Achilles alone, but further investigation is required to confirm this. There is a need for further research regarding this topic in the form of sufficiently powered randomised controlled trials, utilizing validated outcome measures for the measurement of functional changes, blinded assessors and with both medium and long-term follow up.

## Competing interests

The authors declare that they have no competing interests.

## Authors' contributions

DS contributed to the literature search, data extraction, analysis and drafting of the manuscript. BP contributed to the literature search, data extraction, analysis and drafting of the manuscript. LH contributed to the literature search, data extraction, analysis and drafting of the manuscript. RC contributed to the data extraction, analysis and drafting of the manuscript. All authors read and approved the final manuscript.
